# Parallelism of the Eruptive Fevers

**Published:** 1881-03

**Authors:** M. Jules Simon


### PARALLELISM OF THE ERUPTIVE FEVERS.

Abstract of a Lecture delivered at the Hospital des Enfants Malades, Paris,

BY M. JULES SIMON.

The diagnosis of the eruptive fevers is sometimes easy, even at the outset of the disease; in other cases it is impossible, even after the eruption has commenced to appear.

There are certain points of resemblance between them all; all are contagious; smallpox, varioloid and measles can be directly inoculated; scarlet fever is contageous principally through the pellicules of eperdermis which become detached towards the tenth or twelfth day of the disease.

*Measles.*—Measles commences by a chill; there is cephalalgia and vomiting, as in the other fever, but the temperature does not mount suddenly; it gradually becomes higher, to attain its maximum at the moment the eruption makes its appearance. At the same time there supervene symptoms of bronchitis, pharyngitis, accompanied by a hoarse cough, coryza and redness of the conjunctiva, with hypersecretion of tears.

These prodromata exist three or four days before it is possible to establish a diagnosis.

*Variola.*—The outset of smallpox is violent and the fever intense from the beginning, the ascending line of temperature is not vascillating, as in measles; in twenty-four hours it is at its maximum.

The chill is very violent; the fever, more intense than in measles, is, nevertheless, less marked than in scarlatina.

The symptoms, instead of pointing towards the respiratory organs, are rather those of cerebro spinal congestion; intense cephalalgia and rachialgia, sometimes accompanied by a sort of paraplegia.

Sometimes a form of erythema, the variolous rash, appears, preferably in the groins, simulating the rash act of scarlatina.

There is often, too, vomiting and convulsions, new sources of error in forming a diagnosis; the prodromata in definitive cases last two or three days.

*Scarlatina.*—Scarlatina comes on very suddenly; the fever is moderate, or, on the other hand, very intense. This peculiarity explains why certain physicians, judging, undoubtedly, from a limited number of cases, have considerered this a benign malady; while for others it is the most fatal of all diseases.

It is thus that Tissot has compared the disease to dogs who bite without barking. In the great majority of cases the fever rapidly bocomes very violent, the temperature attaining 105° or 106°; a temperature met with in no other disease with the exception of typhoid fever. But during this period the patient in scarlitina suffers from the throat, and on examination the fauces, the tonsils are seen to be scarlet red; white spots soon commence to appear on the tonsils, while the rest of the pharyngeal mucous membrane appears of a dark red color.

The glands about the throat are simultaneously affected; and finally all the symptoms last but twenty-four to thirty-six hours when the eruptions appears.

We will return to the consideration of measles; we hear it every day repeated, that the eruption appears first on the face and neck; but in children it is not in this situation that it must be sought for; in them it appears first behind the ears, or on the back.

The eruption is characterized by small red spots (*taches*), which are often papular, and as the tissues of the young child become easily affected with œdema, if the fever is intense, and the child then seen for the first time, it may easily be mistaken for a case of smallpox.

In such a case it is better to defer for two or three days the expression of a positive opinion.

During this early period the prodromic symptoms of measles, coryza, conjuntivitis, etc., instead of disappearing, become more marked.

In connection with scarlatina, of which the diagnosis is often easy, it must be remembered that cases are met with in which there is very little fever or eruption.

Children may be brought to you in the tenth or twelfth day of the disease; there remains no trace of the scarlet rash; small brownish spots are found, which are the seat of epithelial desquamation; this last sign must be carefully sought for, particularly on the back and in the armpits.

The eruption in smallpox occurs on the face and hands and on the lower limbs. It consists of papules, which on the third day are surmounted by a vesicle, which becomes umbilicated, and the liquid contained therein purulent; dessication occurs about the fifteenth day.

On the eighth day, that is, at the moment of suppuration, the temperature, which had fallen, becomes almost as high as at the beginning of the disease (secondary fever). In varioloid dessication commences after the sixth day, and there is no secondary fever, as in variola.

It is, nevertheless, sometimes very difficult to distinguish between the two, particularly when the eruption in variola is confluent.

Smallpox is, as we have observed, accompanied by a rash, which may simulate a scarlatiniform eruption; the same happens in certain rare cases of diphtheria.

As a general rule, the eruption in scarlatina is found on the neck, in the armpits, the groins, in points where the skin is very delicate. The redness is diffused, and more marked or scarlet in certain points, containing with the color of the skin on neighboring parts, all being covered with sudamina.

The fever lasts eight or ten days, and when it falls, retrocedes gradually.

In all cases convalescence is very long and dangerous because the least imprudence may become the starting point of complications of exceeding gravity.

About the twelfth day albumen is found in the urine, and if the child take cold a very grave attack of angina or fatal nephritis may supervene.

It must not be thought, because fever is no longer present and desquamation is taking place, that the malady is at an end. The greatest care must be taken of the child, in order to prevent his taking cold; the room he is in should not be freely ventilated for a month; his skin is excessively sensitive, and a fetid atmosphere is better than a free supply of air, which, though pure, is cold. We should remember that, in ninety cases out of a hundred, just such imprudence has been the means of leading the little patient to the tomb.

[illegible] conclusion, after an attack of measles, the little patient should remain at least a month confined to the house; a period of two months is not too much after scarlatina and smallpox.—*Medical and Surgical Reporter.*

